# Genetic association of NOS3 rs1799983 variant and hypertension across multiple rural Indonesian populations: a multi-ethnic population-based study

**DOI:** 10.3389/fcvm.2026.1829300

**Published:** 2026-09-23

**Authors:** Bambang Dwiputra, Widyan Putra Anantawikrama, Bambang Widyantoro, Ade Meidian Ambari, Jason Phowira, Naufal Zuhdi, Sri Idaiani, Ari Winasti Satyagraha, Reza Yuridian Purwoko, Elok Ekawati, Shoma Adhi Wijaya, Anwar Santoso

**Affiliations:** 1 Department of Cardiology and Vascular Medicine, Faculty of Medicine, Universitas Indonesia – National Cardiovascular Center Harapan Kita, Jakarta, Indonesia; 2 Research Center for Preclinical and Clinical Medicine, National Research and Innovation Agency, Jakarta, Indonesia; 3 Eijkman Research Center for Molecular Biology, National Research and Innovation Agency, Jakarta, Indonesia; 4 Division of Cardiovascular Research and Development, National Cardiovascular Center Harapan Kita, Jakarta, Indonesia

**Keywords:** genetics, Hypertension, *NOS3* gene, rs1799983 variant, rural

## Abstract

**Introduction:**

Hypertension is a complex polygenic disorder where genetic variants become clinically relevant primarily when they contribute to endothelial dysfunction. While the *NOS3* rs1799983 variant is known to impair endothelial function, data regarding its genotypic and allelic frequency and association with hypertension in Indonesian rural populations remains limited. The present study aimed to investigate the genotypic and allelic frequency of *NOS3* rs1799983 and examine its association with hypertension across multiple ethnic groups in rural Indonesian populations.

**Materials & methods:**

This population-based case-control study included 798 individuals with hypertension and 448 subjects with normal blood pressure from four primarily rural Indonesian regions: Papua, Pamijahan, Morotai, and Natuna. Genotyping of the *NOS3* rs1799983 variant was performed using real-time polymerase chain reaction. Multiple logistic regression models were employed to evaluate the association between genotypes and hypertension.

**Results:**

In the pooled population, no significant unadjusted categorical association existed between the NOS3 variant and hypertension (*p* = 0.069). Subgroup analysis revealed a nominal unadjusted association in Morotai under the dominant model (OR = 1.675, *p* = 0.017), which lost statistical significance following false discovery rate (FDR) correction (adjusted *p* = 0.051). Numerically, GT + TT carriers exhibited significantly higher mean systolic blood pressure (SBP) in the Morotai normotensive control group, an effect that survived FDR correction (adjusted *p* = 0.016). Conversely, unadjusted SBP elevations among GT + TT carriers within the hypertensive cohorts (overall *p* = 0.024; Morotai *p* = 0.016) became non-significant after FDR correction (adjusted *p* = 0.064). Crucially, after adjusting for confounders using multivariate analysis, the GT + TT genotypes emerged as an independent predictor of increased hypertension risk in both the pooled population (aOR = 1.392, 95% CI: 1.045–1.853, *p* = 0.024) and the Morotai subpopulation (aOR = 2.056, *p* = 0.002).

**Conclusions:**

The T allele of the *NOS3* rs1799983 variant was rare in the studied populations. While no significant association was found between the *NOS3* rs1799983 variant and hypertension overall, a modest association was observed under a dominant model specifically within the rural Morotai population. Further research incorporating physiopathological assessments of endothelial function is required to definitively link this variant to hypertension in these rural groups.

## Introduction

Hypertension is a complex polygenic and multifactorial condition that constitutes a major global public health concern, implicated in approximately 8.4 million deaths annually and affecting an estimated 31.1% of the adult population worldwide (1,2). In Indonesia, epidemiological data from the National Health Survey reveal a progressive escalation in hypertension prevalence, increasing from 25.8% in 2013 to 34.1% in 2018, with projections indicating a sustained upward trajectory (2,3)

Extensive research has explored the genetic basis of hypertension, including variants in genes related to eNOS, AGT M235T (rs699), and ACE I/D have been linked to endothelial dysfunction and RAAS alterations, increasing the risk of hypertension and cardiovascular disease (4). Within the *NOS3* gene, several genetic variants are highly relevant in the context of hypertension and other chronic degenerative diseases, such as type 2 diabetes and chronic kidney disease. Specifically, the 4b/a variable number of tandem repeats (VNTR) in intron 4 and the -T786C (rs2070744) variant in the promoter region significantly reduce basal nitric oxide bioavailability and impair transcriptional activity, potentially contributing to endothelial dysfunction (5,6). Notably, *NOS3* rs1799983, one of the most studied hypertension-related genes, has shown varying associations across populations (7–10).

Hypertension genetic susceptibility in Indonesia, particularly rural area, has remained unexplored and absent in databases such as 1000 Genome and gnomAD (11,12). Research on the *NOS3* variant in Indonesian populations also remains limited, highlighting the need for further investigation into its role in hypertension. The study of isolated, rural communities can sometimes reveal localized genetic associations that are otherwise masked in larger, mixed urban cohorts.

Therefore, the primary objective of this study was to establish a crucial genetic baseline by characterizing the genotypic and allelic frequency of the *NOS3* rs1799983 variant across multiple geographically isolated, rural Indonesian populations (Morotai, Natuna, Pamijahan, and Papua). As a secondary objective, we aimed to examine the preliminary association of this variant with hypertension within these unique multi-ethnic cohorts to generate localized, hypothesis-driving data for future cardiovascular research.

## Materials and methods

### Case and control subjects

This population-based case-control study had received approval from the ethics committee, Institutional Review Board of National Cardiovascular Center Harapan Kita. To ensure a representative sample of the rural Indonesian population, we collected data from four distinct rural regions: Jayapura, Pamijahan, Natuna Island, and Morotai Island (from September 2015 to July 2023). The detailed participant recruitment, screening, and selection process is illustrated in the study flow diagram (Figure 1). Participants were recruited consecutively from individuals identified during house-to-house screenings conducted by health officers. These visits included the administration of a standardized questionnaire and assessments of blood pressure, waist circumference, and body mass index (BMI). On the day of sampling, all participants underwent a follow-up examination by healthcare professionals. Eligible subjects were adults (≥18 years) who provided informed consent. Because recruitment utilized convenience sampling during health screenings with an intentional oversampling of cases to maximize the power of genetic testing, the resulting case-to-control ratios in this study do not represent actual regional hypertension prevalences.

**Figure 1:**
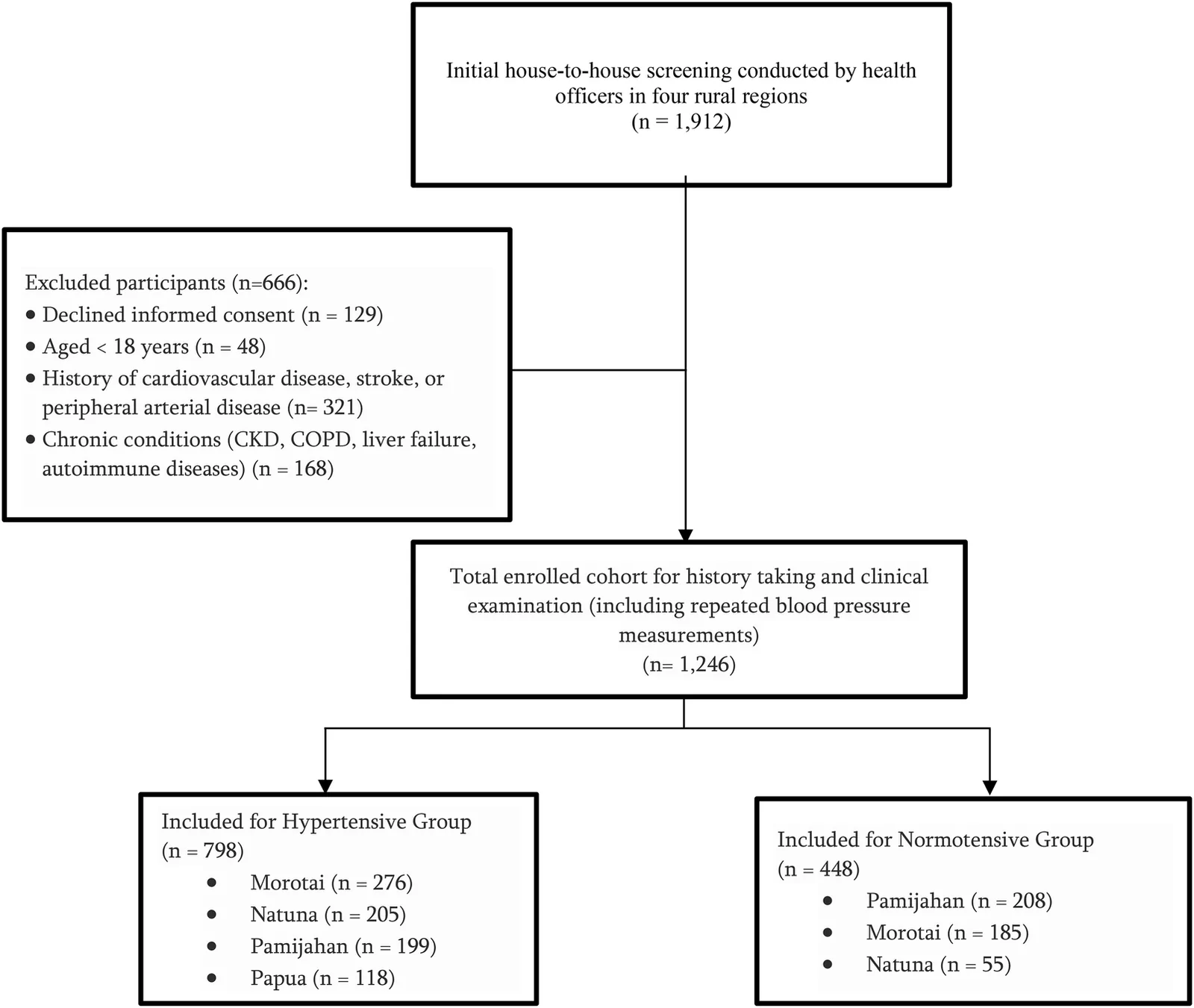
Study flow diagram of participant recruitment and selection.

To strictly ensure the accuracy of clinical phenotypes, blood pressure was measured using a validated automated digital sphygmomanometer. Participants were required to sit quietly in a rested state for a minimum of 10 min prior to evaluation. Blood pressure was measured three consecutive times with a 1–2-min interval between each reading. To prevent white-coat effects or transient spikes, the first reading was discarded, and the average of the second and third readings was calculated to definitively classify individuals as either normotensive or hypertensive. Hypertension was defined as clinically treated hypertension or repeated systolic blood pressure ≥140 mmHg and/or diastolic blood pressure ≥90 mmHg in the absence of antihypertensive medication. Exclusion criteria included symptomatic peripheral arterial disease, stroke, a known history of cardiovascular disease, and other chronic conditions such as chronic kidney disease, chronic obstructive pulmonary disease, chronic liver failure, and autoimmune diseases. To ensure the genetic integrity of our subpopulations, ethnicity was ascertained through stringent self-reporting criteria. To minimize the confounding effects of recent genetic admixture, participants were required to explicitly confirm that both of their parents and at least three out of four of their grandparents belonged to the same ethnic group. The diagnoses of type 2 diabetes mellitus (T2D) and dyslipidemia were established based on one or more of the following criteria: a documented prior physician diagnosis, the current active use of prescribed anti-diabetic or lipid-lowering medications, or abnormal point-of-care fasting blood glucose and lipid profile measurements obtained during the field screening.

### DNA sample preparation and genotyping

A 6 mL sample of venous blood was collected from each participant, followed by immediate isolation of peripheral blood mononuclear cells (PBMCs). The isolated PBMCs were preserved at −80 °C to ensure sample integrity during transport and storage. Genomic DNA was extracted from peripheral blood leukocytes utilizing the Geneaid Nucleic Acid Extraction Kit (Catalog No. GB300; Geneaid Biotech Ltd., New Taipei City, Taiwan) in strict accordance with the manufacturer's established protocols. Genotyping of the *NOS3* rs1799983 variant was subsequently performed using real-time polymerase chain reaction (qPCR). The amplification and allelic discrimination were executed utilizing the TaqMan™ genotyping system with Applied Biosystems reagents (Catalog No. 4346906; Applied Biosystems, Waltham, MA, USA). The specific TaqMan SNP Genotyping Assay utilized was C___3219460_20. The overall genotyping call rate achieved was 100% and strict analytical quality control was maintained through duplicate sample concordance checking. Furthermore, all laboratory personnel were completely blinded to the clinical case/control status of the samples throughout the genotyping procedures.

### Statistical analysis

All statistical analyses were performed using SPSS software for Mac (version 29.0, IBM Corp.). The Hardy-Weinberg equilibrium (HWE) was assessed specifically within the normotensive control groups of the pooled population and each respective subpopulation using a chi-square goodness-of-fit test. A *P*-value ≥0.05 was considered to indicate adherence to HWE. Differences in genotype and allele frequencies between hypertensive and normotensive groups were assessed using the chi-square (*χ*^2^) test, and associations were quantified by calculating odds ratios (ORs) with corresponding 95% confidence intervals (CIs). Genotypic associations were further examined under autosomal dominant model of inheritance using the *χ*^2^test, and ORs were computed accordingly. The genotype and allele frequencies across all models (allelic, additive, dominant, and recessive) are presented in Table 3. Because the TT genotype (homozygous TT genotype) was rare across the studied rural populations, the recessive model was statistically underpowered. Therefore, our primary analysis and subsequent physiological assessments focused on the dominant model (GG vs. GT + TT) to evaluate the impact of carrying at least one minor T allele.

Binary logistic regression models were employed during genotype-based comparisons to adjust for potential confounders including age, sex, body mass index (BMI), smoking status, DM (Diabetes Mellitus), and dyslipidemia. Furthermore, the influence of demographic and clinical variables (such as age, sex, BMI, smoking habits, and comorbidities like diabetes mellitus and dyslipidemia) on genotype distribution was assessed. One-way analysis of variance (ANOVA) was used for continuous variables, while categorical variables were analyzed using either the *χ*^2^ test or Fisher's exact test, depending on data distribution. To correct for multiple testing across the three distinct subpopulations (Morotai, Natuna, Pamijahan; *m* = 3), the Benjamini-Hochberg False Discovery Rate (FDR) procedure was applied. This Benjamini-Hochberg False Discovery Rate (FDR) procedure was applied consistently across the primary unadjusted dominant models, the multivariable logistic regression models, and the continuous numerical blood pressure comparisons to strictly control for multiple testing. Adjusted *p*-values were calculated based on the rank of the raw *p*-values within each respective family of tests.

## Results

### Characteristics of the study population

The study included 798 Individuals with hypertension and 448 Subjects with normal blood pressure. The baseline characteristics of the subjects were summarized in Table 1. The average age was 55.73 ± 10.83 years in the hypertensive group and 49.58 ± 12.57 years in controls. Hypertensive individuals were significantly older and had elevated blood pressure and higher BMI compared to controls (*p* < 0.05). A greater proportion of Patients with had comorbid conditions like diabetes and dyslipidemia (*p* < 0.05). Smoking rates did not differ significantly between the case and control groups (24.4% vs. 21.7%, *p* = 0.297).

**Table 1:** Baseline sociodemographic and clinical characteristics of participants according to hypertension status.

Variables	Case (*n* = 798)	Control (*n* = 448)	*P* value
Mean age ± SD	55.73 ± 10.83	49.58 ± 12.57	<0.001
Number of males (percentage)	249 (31.2%)	134 (29.9%)	0.635
Mean systolic BP (mm Hg)	169.47 ± 21.76	119.43 ± 14.55	<0.001
Mean diastolic BP (mm Hg)	97.09 ± 14.59	74.52 ± 8.25	<0.001
BMI	26.86 ± 5.15	24.94 ± 5.07	<0.001
Waist Circumference (cm)	91.07 ± 12.08	85.02 ± 12.46	<0.001
Smoking	195 (24.4%)	97 (21.7%)	0.266
Diabetes	98 (12.3%)	32 (7.1%)	0.004
Dyslipidemia	149 (18.7%)	22 (4.9%)	<0.001
Ethnicity distribution			<0.001
Sundanese	202 (25.4%)	223 (49.8%)	
Galela/Tobelo	204 (25.6%)	67 (14.9%)	
Malay	193 (24.2%)	52 (11.6%)	
Papuan	64 (8.0%)	0 (0%)	
Bugis	27 (3.4%)	26 (5.8%)	
Javanese	16 (2.0%)	23 (5.1%)	
Others	92 (11.5%)	57 (12.7%)	

*P*-values were obtained using the *χ*^2^ test for categorical variables (gender, smoking, diabetes, stroke, myocardial infarction, dyslipidemia), Fisher's exact test for categorical variables not suitable for the *χ*^2^ test (renal failure), and the independent sample t-test for continuous variables (age, BMI, and systolic and diastolic blood pressure).

The study population reflected the rich ethnic diversity of the Indonesian archipelago. This diversity is broadly characterized by a division between western Indonesia, which is dominated by groups of Austronesian ancestry like the Javanese, Sundanese, and Malay, and eastern Indonesia, where populations of mixed Melanesian ancestry such as the Papuan and Galela/Tobelo are predominant. Our cohort captured this distribution effectively. The three most represented ethnic groups were Sundanese (*n* = 425; 34.1%), Galela/Tobelo (*n* = 271; 21.8%), and Malay (*n* = 245; 19.7%). Other notable groups included Papuan (*n* = 64; 5.1%), Bugis (*n* = 53; 4.3%), and Javanese (*n* = 39; 3.1%). The remainder of the cohort (*n* = 149; 11.9%) consisted of various other ethnicities. The geographical distribution, native ancestries, and baseline *NOS3* rs1799983 genotype frequencies (GG, GT, TT) across the four isolated rural regions are geographically visualized in Figure 2.

**Figure 2:**
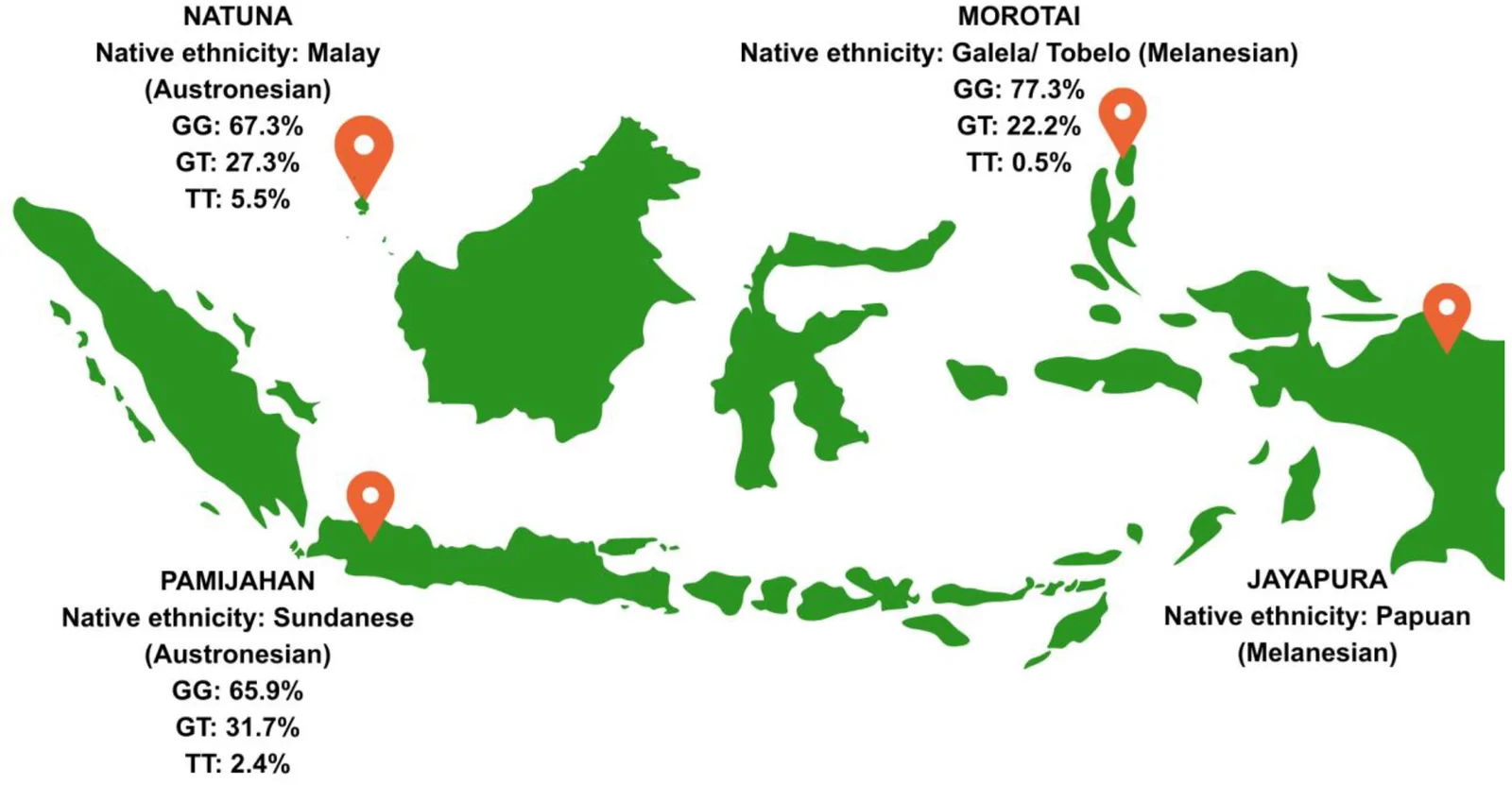
Geographic distribution and genetic ancestry of the study populations across the Indonesian archipelago. The map highlights the four isolated rural regions sampled in this study (Natuna, Pamijahan, Morotai, and Jayapura), illustrating the longitudinal Asian-Melanesian genetic ancestry cline. Baseline genotype frequencies (GG, GT, and TT) of the *NOS3* rs1799983 variant are provided for each respective population.

The genotype distributions among the normotensive control groups in the Morotai (*p* = 0.283), Natuna (*p* = 0.385), and Pamijahan (*p* = 0.367) subpopulations, did not deviate significantly from Hardy-Weinberg equilibrium, confirming the stability and representative nature of the sampled cohorts. In Morotai, the GG genotype was more frequent in subjects with normal blood pressure (77.3%) compared to patients with hypertension (67.0%). The GT genotype was more common among patients with hypertension (30.8%). No significant associations were found in Natuna and Pamijahan. In the aggregated Indonesian population, the GG genotype was predominant in both hypertensive (67.5%) and control groups (70.8%) (Table 2).

**Table 2:** Genotypic distribution of the *NOS3* rs1799983 variant among the study populations.

Genotype	Case (*n* = 798)	Control (*n* = 448)	*P* value
Genotype distribution (all population)			
GG	539 (67.5%)	317 (70.8%)	
GT	236 (29.6%)	122 (27.2%)	
TT	23 (2.9%)	9 (2.0%)	
Morotai	Case (*n* = 276)	Control (*n* = 185)	HWE (Controls): *p* = 0.283
GG	185 (67.0%)	143 (77.3%)	
GT	85 (30.8%)	41 (22.2%)	
TT	6 (2.2%)	1 (0.5%)	
Natuna	Case (*n* = 205)	Control (*n* = 55)	HWE (Controls): *p* = 0.385
GG	138 (67.3%)	37 (67.3%)	
GT	61 (29.8%)	15 (27.3%)	
TT	6 (2.9%)	3 (5.5%)	
Pamijahan	Case (*n* = 199)	Control (*n* = 208)	HWE (Controls): *p* = 0.367
GG	123 (61.8%)	137 (65.9%)	
GT	69 (34.7%)	66 (31.7%)	
TT	7 (3.5%)	5 (2.4%)	
Papua	Case (*n* = 118)	—	—
GG	93 (78.8%)	—	
GT	21 (17.8%)	—	
TT	4 (3.4%)	—	

**Table 3:** Categorical analysis of association between *NOS3* rs1799983 and hypertension in all- and sub-group population.

Analysis group	Genotype group	Case n(%)	Control n(%)	Statistical analysis
Overall population	GG	446 (65.6%)	317 (70.8%)	*χ*^2^ = 3.299 *p* = 0.069 OR (95% CI) = 1.270 (0.981–1.643)
	GT + TT	234 (34.4%)	131 (29.2%)	
Morotai	GG	185 (67.0%)	143 (77.3%)	*χ*^2^ = 5.689 *p* = 0.017 OR (95% CI) = 1.675 (1.094–2.56)
	GT + TT	91 (33.0%)	42 (22.7%)	
Natuna	GG	138 (67.3%)	37 (67.3%)	*χ*^2^ = 0.000 *p* = 0.995 OR (95% CI) = 0.998 (0.529–1.88)
	GT + TT	67 (32.7%)	18 (32.7%)	
Pamijahan	GG	123 (61.8%)	137 (65.9%)	*χ*^2^ = 0.725 *p* = 0.394 OR (95% CI) = 1.192 (0.795–1.79)
	GT + TT	76 (38.2%)	71 (34.1%)	

* Fisher's exact test was done due to unsuitability to chi-square test.

### Categorical analysis

Under the dominant model, no significant association was observed between the NOS3 rs1799983 variant and hypertension in the pooled population (OR = 1.270; 95% CI: 0.981–1.643, *p* = 0.069). In subgroup analysis, Morotai showed a nominal unadjusted association under the dominant model (*p* = 0.017), with GT + TT carriers showing higher odds of hypertension than GG carriers (OR = 1.675; 95% CI: 1.094–2.56). In contrast, no significant associations were observed in the Natuna or Pamijahan subpopulations under this model. In Morotai, the three genotype comparison (GG vs. GT vs. TT) also showed a nominal difference between patients with hypertension and normotensive controls (*p* = 0.037). Because the TT genotype was rare, the dominant model (GG vs. GT + TT) was prespecified as the primary genetic model for association testing. After Benjamini-Hochberg false discovery rate correction for multiple subgroup testing, the Morotai finding did not retain statistical significance (adjusted *p* = 0.051, Table 7) and should be interpreted as hypothesis-generating rather than definitive. The odds ratios and 95% confidence intervals for the dominant model across all cohorts are visually summarized in Figure 3.

**Figure 3:**
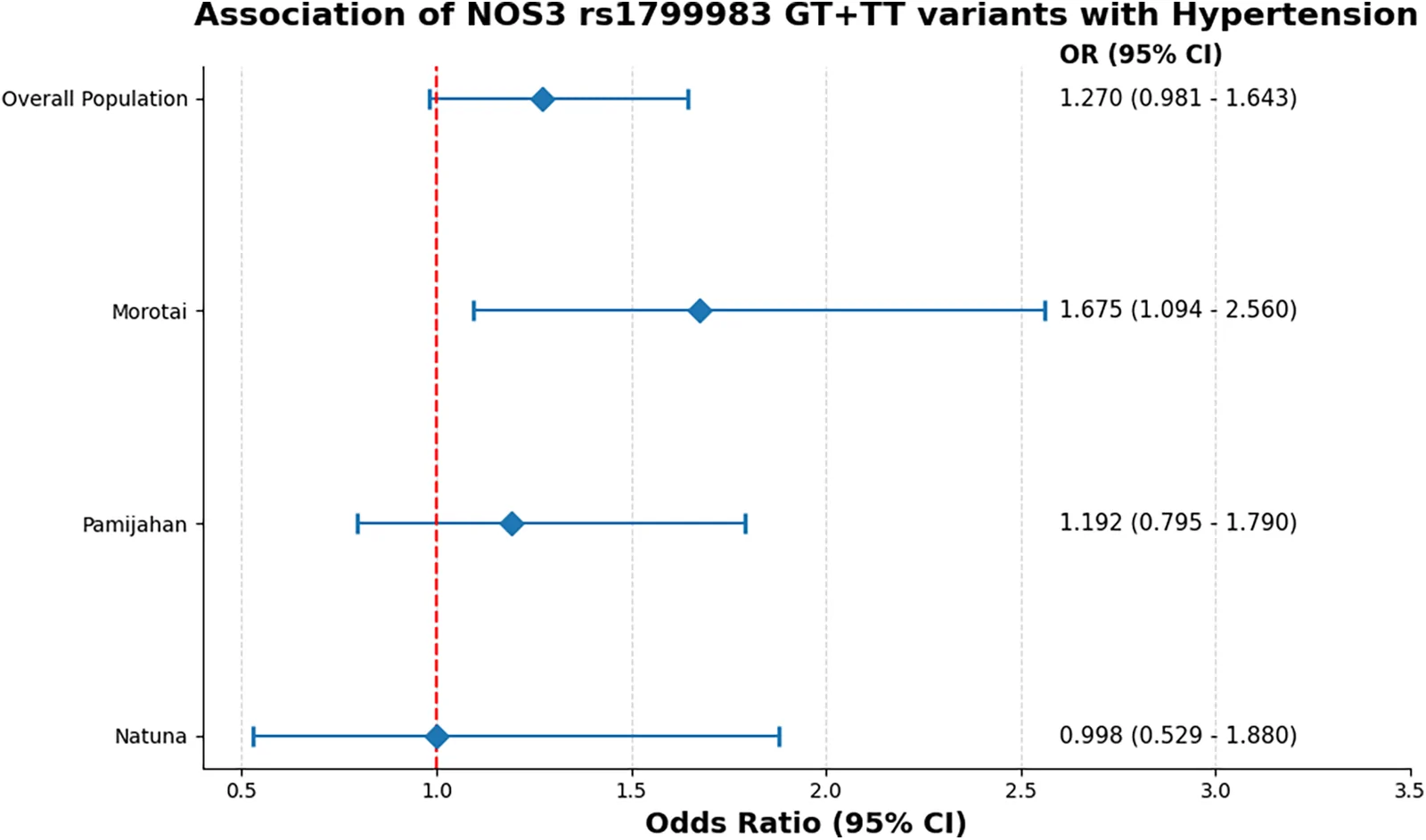
Forest plot of the association between the *NOS3* rs1799983 variant and hypertension risk. Data represent the Dominant Model (GT + TT vs. GG genotypes). The plot displays the Odds Ratios (OR) and 95% Confidence Intervals (CI) for the pooled overall population and individual rural subgroups. The dashed red line indicates no effect (OR = 1). A nominal unadjusted association with increased hypertension odds was observed in the Morotai population, but this finding should be interpreted as hypothesis generating after correction for multiple subgroup testing.

### Numerical analysis

To eliminate confounding caused by combining case and control cohorts, the physiological impact of the NOS3 variant on systolic blood pressure (SBP) was analyzed separately within the normotensive and hypertensive groups (Table 4). Among healthy normotensive controls, no statistically significant differences in SBP were observed between genotypes in the overall population (*p* = 0.453). However, mutant T-allele carriers in the Morotai control group exhibited a significantly higher mean SBP (127.78 vs. 119.88 mmHg, *p* = 0.002) (Figure 4), an elevation that survived FDR correction (adjusted *p* = 0.016; Table 8).

**Figure 4:**
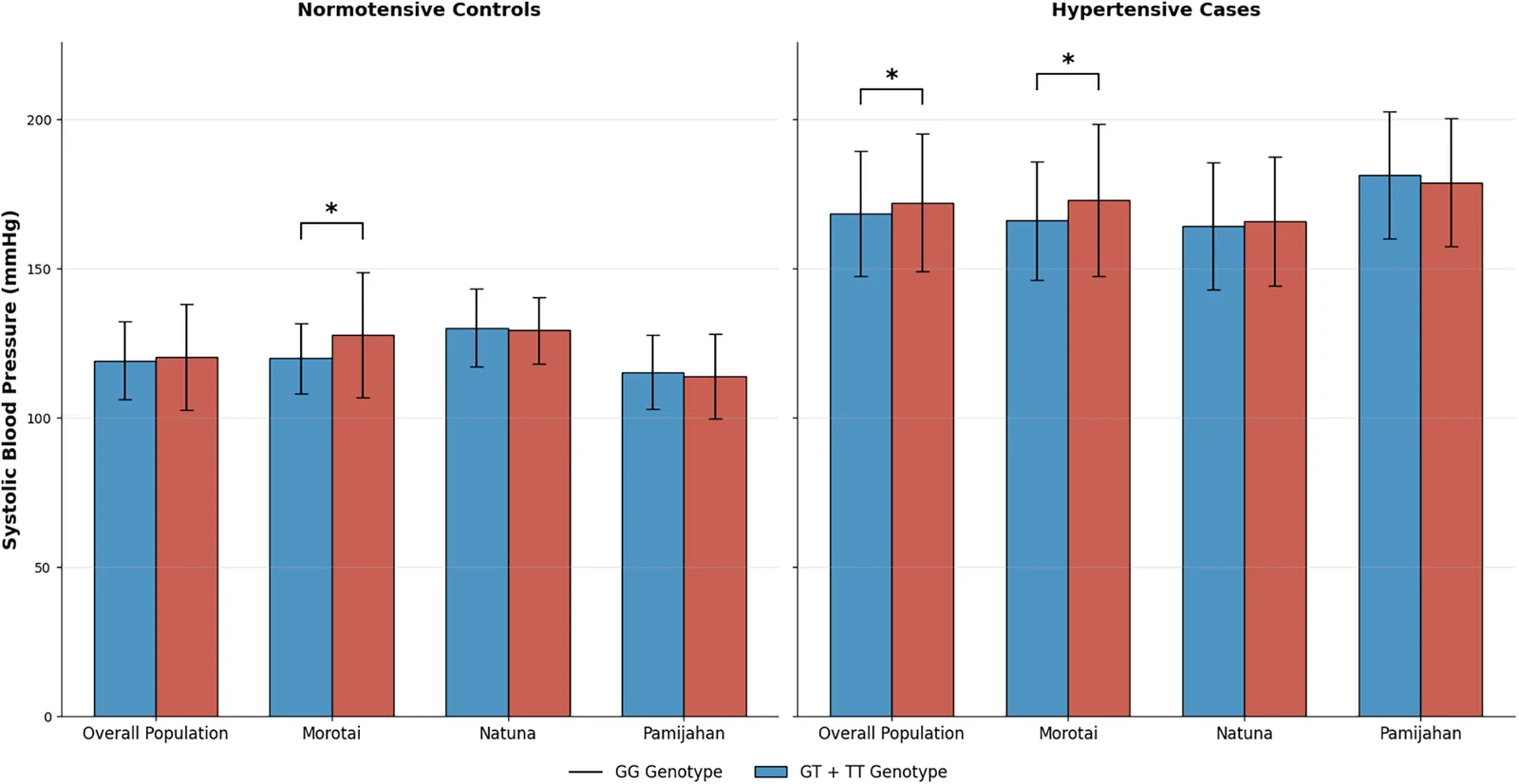
Comparison of systolic blood pressure by *NOS3* rs1799983 genotype across study populations. The grouped bar charts illustrate mean systolic blood pressure (mmHg) for individuals carrying the GG genotype (blue) versus the GT + TT genotypes (red). The cohort was stratified to isolate normotensive controls (left panel) from hypertensive cases (right panel) to prevent confounding. Error bars represent standard deviations. Groups carrying the T allele showed a significant elevation of systolic blood pressure within the Morotai normotensive control population, as well as in the Morotai and pooled hypertensive populations. Significant differences are denoted by asterisks (* *p* < 0.05).

**Table 4:** Numerical analysis of association between *NOS3* rs1799983 and hypertension in all- and sub-group population.

Dominant model (GG vs. GT + TT)						
Variable	Population	HT status	GG	GT + TT	Mean Difference (95%CI)	*P*-value
SBP	Overall population	Control Case	119.04 ± 13.01 168.27 ± 21.03	120.38 ± 17.76 171.98 ± 23.07	−1.35 (−4.32 to 1.62) −3.72 (−6.94 to −0.49)	0.373 0.024
	Morotai	Control Case	119.88 ± 11.80 165.92 ± 19.84	127.78 ± 20.94 172.73 ± 25.51	−7.90 (−12.91 to 2.89) −6.81 (−12.35 to −1.27)	0.002 0.016
	Natuna	Control Case	130.05 ± 13.03 164.07 ± 21.26	129.15 ± 11.19 165.72 ± 21.55	0.91 (−6.28 to 8.09) −1.65 (−7.92 to 4.62)	0.801 0.605
	Pamijahan	Control Case	115.18 ± 12.41 181.18 ± 21.36	113.79 ± 14.20 178.75 ± 21.53	1.39 (−2.37 to 5.16) 2.43 (−3.74 to 8.59)	0.466 0.439
DBP	Overall population	Control Case	74.86 ± 8.52 96.47 ± 14.75	74.74 ± 9.58 98.20 ± 14.39	0.12 (−1.69 to 1.92) −1.73 (−3.90 to 0.44)	0.899 0.119
	Morotai	Control Case	74.68 ± 8.47 90.90 ± 14.53	74.98 ± 9.96 91.85 ± 14.88	−0.30 (−3.36 to 2.75) −0.96 (−4.65 to 2.73)	0.845 0.610
	Natuna	Control Case	80.59 ± 8.05 95.54 ± 12.56	77.65 ± 7.20 97.27 ± 12.86	2.95 (−1.54 to 7.44) −1.73 (−5.45 to 1.98)	0.194 0.359
	Pamijahan	Control Case	73.29 ± 7.76 108.39 ± 13.58	73.86 ± 9.84 107.94 ± 11.30	−0.56 (−3.02 to 1.89) 0.44 (−3.23 to 4.12)	0.651 0.812

DBP, diastolic blood pressure; SBP, systolic blood pressure.

In stark contrast, when analyzed strictly within the hypertensive case cohort, carrying the mutant T allele was associated with a significant physiological exacerbation of blood pressure severity. In the overall hypertensive population, GT/TT carriers exhibited a significantly higher mean SBP compared to GG homozygotes (171.98 vs. 168.27 mmHg, *p* = 0.024). This physiological exacerbation was most pronounced within the Morotai hypertensive cohort, where the presence of the T allele drove systolic pressure an average of 6.81 mmHg higher than the wild-type baseline (*p* = 0.016). However, it is important to note that after applying a secondary Benjamini–Hochberg false discovery rate correction across these continuous blood pressure comparisons, this physiological elevation became non-significant (adjusted *p* = 0.064; Table 8). These stratified results suggest that while the variant may not independently induce elevated blood pressure in healthy controls, it may exert a modulatory effect that exacerbates systolic severity once the hypertensive phenotype is established.

### Multivariate analysis

The multivariate analysis using binary logistic regression identified several risk factors for hypertension. In pooled rural Indonesian population, advancing age, higher BMI, and dyslipidemia were significantly associated with an increased risk of hypertension. Each additional year of age increased the likelihood of hypertension by 6.1% (aOR = 1.061; *p* < 0.001), and individuals with elevated BMI had a higher risk (aOR = 1.091; *p* < 0.001). Dyslipidemia was found to be the strongest predictor, with a 5.16-fold increased risk (aOR = 5.161; *p* < 0.001). The analysis revealed that in the pooled overall population (excluding Papua), carrying the mutant T allele (GT + TT genotypes) was an independent predictor of hypertension (aOR = 1.392; 95% CI: 1.045–1.853; *p* = 0.024) after adjusting for all clinical confounders. This effect was most distinctly localized within the Morotai subpopulation. In Morotai, carriers of the mutant T allele demonstrated a significantly increased independent risk of hypertension compared to GG homozygotes, even after adjusting for all clinical confounders (aOR = 2.056; 95% CI: 1.290–3.276; raw *p* = 0.002; FDR-adjusted *p* = 0.006). In the Morotai population, age (aOR = 1.045; *p* < 0.001), BMI (aOR = 1.064; *p* = 0.002), and smoking (aOR = 2.163; *p* = 0.008) were also significant predictors of hypertension. In Natuna, age (aOR = 1.048; *p* = 0.003) and dyslipidemia (aOR = 2.494; *p* = 0.009) were significant factors, but the *NOS3* rs1799983 variant did not correlate with hypertension. In Pamijahan, advancing age (aOR = 1.106; *p* < 0.001), elevated BMI (aOR = 1.200; *P* < 0.001), and dyslipidemia (aOR = 23.584; *p* = 0.003) were major risk factors, though the *NOS3* variant showed no significant association with hypertension in this group (Table 5).

**Table 5:** Multivariate logistic regression analysis of risk factors for hypertension in all- and sub-group population.

Analysis group	Variable		aOR	aOR (95%CI)	*P*-value
Pooled population	Age		1.061	1.049–1.074	<0.001
	Gender	Female	1		0.849
		Male	0.966	0.676–1.361	
	BMI		1.091	1.062–1.122	<0.001
	Smoking	No Smoking	1		
		Smoking	1.454	0.986–2.143	0.059
	DM	No DM	1		
		DM	1.453	0.918–2.302	0.111
	Dyslipidemia	No Dyslipidemia	1		
		Dyslipidemia	5.161	3.181–8.373	<0.001
	rs1799983	GG	1		
		GT + TT	1.392	1.045–1.853	0.024
Morotai	Age		1.045	1.029–1.061	<0.001
	Gender	Female	1		
		Male	1.082	0.662–1.770	0.752
	BMI		1.064	1.023–1.108	0.002
	Smoking	No Smoking	1		
		Smoking	2.163	1.228–3.809	0.008
	DM	No DM	1		
		DM	1.585	0.833–3.019	0.161
	Dyslipidemia	No Dyslipidemia	1		
		Dyslipidemia	1.718	0.545–5.423	0.356
	rs1799983	GG	1		
		GT + TT	2.056	1.290–3.276	0.002
Natuna	Age		1.048	1.016–1.080	0.003
	Gender	Female	1		
		Male	0.704	0.306–1.619	0.409
	BMI		0.972	0.907–1.042	0.428
	Smoking	No Smoking	1		
		Smoking	0.521	0.223–1.215	0.131
	DM	No DM	1		
		DM	0.805	0.328–1.975	0.636
	Dyslipidemia	No Dyslipidemia	1		
		Dyslipidemia	2.494	1.261–4.931	0.009
	rs1799983	GG	1		
		GT + TT	1.025	0.517–2.033	0.943
Pamijahan	Age		1.106	1.075–1.137	<0.001
	Gender	Female	1		
		Male	1.131	0.527–2.430	0.751
	BMI		1.200	1.137–1.266	<0.001
	Smoking	No Smoking	1		
		Smoking	1.387	0.605–3.179	0.439
	DM	No DM	1		
		DM	1.117	0.386–3.234	0.839
	Dyslipidemia	No Dyslipidemia	1		
		Dyslipidemia	23.584	2.960–187.887	0.003
	rs1799983	GG	1		
		GT + TT	1.235	0.763–1.999	0.390

## Discussion

This study revealed that in the combined rural Indonesian population cohort, no statistically significant association was observed between the *NOS3* rs1799983 variant and hypertension. In the Morotai population, GT and TT carriers showed a nominal unadjusted association with hypertension under the dominant model; however, this finding did not remain definitive after correction for multiple subgroup testing and should therefore be interpreted as hypothesis generating. Multivariate analysis revealed that after adjusting for other confounding risk factors, the NOS3 rs1799983 variant was independently associated with an increased risk of hypertension in the pooled population.

To contextualize our findings within the global genetic landscape, the minor T-allele frequency of our overall normotensive control cohort (15.6%) was compared against the 1000 Genomes Project Phase 3 database (Table 6). The Indonesian baseline frequency aligns closely with the South Asian (16.8%) and East Asian (13.0%) populations, while remaining significantly lower than European cohorts (34.4%). This comparative validation not only confirms the genetic consistency of our sampling but underscores the critical importance of our data in filling a prominent representation gap for isolated Southeast Asian populations within major global genomic repositories (11).

**Table 6:** Comparison of *NOS3* rs1799983 variant (T-allele) frequencies between the Indonesian cohort and global super-populations.

Population	T-Allele frequency (%)	Reference
Overall Indonesian Controls	15.6%	Present Study
East Asian (EAS)	13.0%	1000 Genomes Phase 3
South Asian (SAS)	16.8%	1000 Genomes Phase 3
African (AFR)	7.0%	1000 Genomes Phase 3
Admixed American (AMR)	21.5%	1000 Genomes Phase 3
European (EUR)	34.4%	1000 Genomes Phase 3
Global Average	17.6%	1000 Genomes Phase 3

**Table 7:** Benjamini-Hochberg false discovery rate (FDR) adjustment for NOS3 dominant models across subpopulation.

Analysis Model	Subpopulation	Raw *P*-value	Rank	Adjusted *P*-value ()*
Unadjusted dominant	Morotai	0.017	1	**0**.**051**
	Pamijahan	0.394	2	0.591
	Natuna	0.995	3	0.995
Multivariable regression	Morotai	0.002	1	**0**.**006**
	Pamijahan	0.390	2	0.585
	Natuna	0.943	3	0.943

**Table 8:** Benjamini-Hochberg false discovery rate (FDR) adjustment for numerical blood pressure comparisons.

Variable	Subpopulation	Hypertension Status	Raw P-value	Rank	Adjusted P-value (*)
Systolic BP	Morotai	Control	0.002	1	0.016
	Morotai	Case	0.016	2	0.064
	Overall population	Case	0.024	3	0.064
	Overall population	Control	0.373	4	0.621
	Pamijahan	Case	0.439	5	0.621
	Pamijahan	Control	0.466	6	0.621
	Natuna	Case	0.605	7	0.691
	Natuna	Control	0.801	8	0.801
Diastolic BP	Overall population	Case	0.138	1	0.776
	Natuna	Control	0.194	2	0.776
	Natuna	Case	0.359	3	0.907
	Pamijahan	Control	0.651	4	0.907
	Morotai	Case	0.702	5	0.907
	Morotai	Control	0.731	6	0.907
	Pamijahan	Case	0.811	7	0.907
	Overall population	Control	0.907	8	0.907

To the best of our knowledge, this study represents the first multi-ethnic genetic association analysis of hypertension conducted across diverse, geographically isolated rural populations in Indonesia. The population of the Indonesian archipelago is highly diverse, but still under-characterized genetically. Generally, the genetic distribution is varied between islands, with two major genetic ancestries: Papuan, which is closer to the first anatomically modern humans of Sunda and Sahul, and Asian, which is associated with the Neolithic migration of Austronesian speakers from Taiwan (13). The proportion of these genetic ancestries are longitudinally correlated, with Papuan component being more predominant in the eastern population of the country, reaching its highest frequency in the Papua provinces, but absent west of Wallace's line. Meanwhile, the western part of the archipelago has a more complex admixture of Austronesian ancestries between different islands despite their geographic proximity (14–16). However, while there are extensive studies on the Javanese and Papuan population and their distinctions, information for population stratification of the Riau (Natuna) and Maluku (Morotai) islands are scarce. Nonetheless, these general diversities between regions serve as the basis of this study design that allows the exploration of genetic differences for such a large country.

The Natuna and Pamijahan populations reflect the environmental characteristics typical of western Indonesia. In Natuna, predominantly inhabited by Melayu people, traditional diets are rich in fish and low in processed foods (17). Pamijahan, a predominantly Sundanese region, has traditionally maintained an agrarian diet rich in vegetables and low in sodium (18). Eastern Indonesian populations, including those in Papua and Morotai, exhibit distinct genetic characteristics due to prolonged isolation and adaptation to unique environmental pressures. Notably, the province of Papua has the lowest reported hypertension prevalence in Indonesia at 22.22%, while other regions report higher rates (19). The lower prevalence of hypertension in Papua may be influenced by traditional lifestyles that include high physical activity levels and diets low in processed foods. These environmental factors can modulate genetic predispositions, potentially reducing hypertension risk. However, the high prevalence of undiagnosed hypertension in Indonesia, estimated at 76.3%, suggests that actual rates may be underreported (20). In Morotai, predominantly inhabited by Tobelo and Galela ethnic groups, traditional diets are high in salt due to fish preservation methods. High dietary salt intake has been associated with increased hypertension risk, particularly in coastal communities where salted dry fish is a staple (21). This dietary habit, combined with genetic predispositions, may contribute to higher hypertension prevalence in this region.

Previous studies have shown that ethnicity affects the *NOS3* rs1799983 variant. Some studies found that the *NOS3* rs1799983 G allele is the major allele in Caucasian, Asian, and African American populations (22,23). Recent studies have indicated a racial difference in the allele frequency of other *NOS3* variant, for example the G allele in rs1808593 is a major allele in Caucasians (24), but a minor genotype in the Japanese and Chinese population (25,26). suggesting that the G allele in rs1808593 is a minor allele in the Asian population. Our study showed that the G allele is the major allele in Indonesia and all subpopulations included in this study, which is aligned to previous studies. Our findings showed that in pooled Indonesian population there is no significant association between *NOS3* rs1799983 variant and hypertension which in line with a study by Arjadi et al. in 2015 that found no significant relationship between rs1799983 variant and hypertension, also no relationship between rs1799983 and NO level in Javanese population (27).

However, a meta-analysis conducted by Shi et al. in 2021 showed the association between the eNOS rs1799983 variant and hypertension which provide critical context to our study, particularly in understanding the role of genetic predisposition in varying populations. Their analysis, which included over 14,000 cases and 13,000 controls across multiple populations, demonstrated that the presence of the T allele significantly increases hypertension risk under various genetic models, including dominant and recessive modes of inheritance. This aligns with our findings in the Morotai population, where the GT + TT genotypes of the *NOS3* rs1799983 variant was associated with a 1.675-fold increased risk of hypertension (*p* = 0.017) and higher systolic blood pressure in individuals with GT + TT genotypes. While this unadjusted dominant model did not retain statistical significance after Benjamini-Hochberg false discovery rate correction (adjusted *p* = 0.051), our multivariate analysis revealed a more robust relationship. By adjusting for critical clinical covariates (age, BMI, and smoking status), the independent effect of the mutant T allele in the Morotai population was strengthened (aOR = 2.056). This multivariable association survived FDR correction for multiple testing (adjusted *p* = 0.006). This indicates that within this specific rural population, the genetic risk imparted by the NOS3 variant becomes highly apparent once confounding traditional cardiometabolic factors are controlled for. The previous meta-analysis also showed that the association between genotype and hypertension varied between populations, in which allelic and dominant models showed significant association in Asian populations, but insignificant in non-Asian populations. This is similar to our result that showed significant association in all models in Morotai populations, but not in others.

*NOS3* rs1799983 variant involves a substitution of guanine (G) with thymine (T) at position 894, leading to an amino acid change from glutamate to aspartate in the endothelial nitric oxide synthase (eNOS) enzyme. This substitution is proposed to diminish *NOS3* affinity to caveolin-1, reducing its presence in the caveolar fraction of endothelial cells. Consequently, less *NOS3* is available for activation by calcium-bound calmodulin, leading to decreased *NOS3* activity and reduced nitric oxide (NO) production (28). *NOS3* rs1799983 is also hypothesized to compromise the binding site for the essential cofactor tetrahydrobiopterin (BH4). Consequently, the altered enzyme exhibits an increased susceptibility to proteolytic cleavage, which would lead to decreased *NOS3* activity and significantly reduced nitric oxide (NO) bioavailability (5,6). This diminished NO production may contribute to hypertension through impaired endothelium dependent vasodilation and altered renal vascular regulation. Experimental loss of NOS3 activity has been associated with a hypertensive phenotype, while clinical association studies have reported population specific links between NOS3 rs1799983 and essential hypertension. Therefore, the observed Morotai association should be interpreted as biologically plausible but hypothesis generating, pending direct assessment of endothelial function and renal physiology (29, 30). Consequently, it is proposed that individuals carrying the T allele may exhibit an increased risk of hypertension modulated by this impaired NO-mediated vasodilation and altered renal physiology. While our epidemiological data points to a localized association in the Morotai population, the definitive physiopathological impact of this reduced NO production requires further validation. Future studies must provide direct evidence that this variant associates with impaired endothelial function, ideally assessed by vasodilation in response to endothelial agonists among hypertensive carriers versus non-carriers. Studies have shown that individuals carrying the T allele may have an increased risk of hypertension due to impaired NO-mediated vasodilation (31) While our epidemiological data points to a localized association in the Morotai population, the definitive physiopathological impact of this reduced NO production requires further validation. Future studies must provide direct evidence that this variant associates with impaired endothelial function, ideally assessed by vasodilation in response to endothelial agonists among hypertensive carriers versus non-carriers.

Hypertension is a multifactorial condition influenced by genetic predispositions, physiological changes, and lifestyle factors. Consistent with broader epidemiological data, our multivariate analysis of the pooled Indonesian population showed after risk factors adjustment that older age, higher BMI, and dyslipidemia significantly increased risk of hypertension. While the *NOS3* rs1799983 variant appears to play a localized role in regions like Morotai, it acts synergistically with these traditional cardiometabolic risk factors, particularly age-related arterial stiffness, adiposity-driven sympathetic activation, and lipid-induced endothelial dysfunction to potentially modulate and exacerbate hypertension pathogenesis.

Furthermore, while our multivariate analysis is appropriately adjusted for general dyslipidemia as a confounding variable, the specific biological interaction between dyslipidemia and the *NOS3* variant warrants critical consideration. Dyslipidemia is a well-established driver of vascular oxidative stress, which accelerates the degradation of nitric oxide. In individuals carrying the *NOS3* rs1799983 variant, concurrent dyslipidemia likely acts synergistically to promote endothelial dysfunction. This compounding gene-metabolic interaction could considerably amplify the overall cardiovascular risk beyond the independent effects of either factor alone, underscoring the necessity of detailed lipid-subtype profiling in precision cardiovascular medicine.

### Study limitations

This study has several important limitations. First, its cross-sectional design restricts our ability to establish a definitive causal relationship between the *NOS3* rs1799983 variant and hypertension. Second, the study did not account for key environmental and lifestyle factors known to influence hypertension risk, such as dietary sodium intake, physical activity, routine medication, and stress. We were unable to comprehensively quantify specific environmental and lifestyle exposures, most notably dietary sodium intake. Given that high-salt diets are culturally prevalent in these coastal archipelagic communities, it is highly plausible that high dietary sodium interacts synergistically with the *NOS3* rs1799983 variant to exacerbate endothelial impairment and the hypertensive phenotype. Future longitudinal studies integrating detailed nutritional epidemiology are required to fully uncouple these interactions. Third, our study focused solely on a single genetic variant (*NOS3* rs1799983), potentially overlooking other vital genetic influences. Fourth, due to logistical and time constraints encountered during the fieldwork in Jayapura, we were unable to successfully recruit a matched normotensive control group for this specific population. Consequently, individuals of Papuan descent were strictly excluded from all categorical case-control genetic association analyses to prevent statistical skewing. However, their inclusion in the broader sociodemographic baseline remains valuable for mapping the overarching epidemiological landscape of these remote archipelagic regions. While a localized association was observed in Morotai, tests for heterogeneity across the regional subgroups showed Cochran's *Q p* of 0.335. Therefore, the Morotai finding currently rests on a difference in significance between subgroups rather than a proven divergent population effect, and must be viewed as an isolated hypothesis-generating observation. Finally, we lacked functional physiopathological data; measuring endothelial function (e.g., vasodilation in response to agonists) is required in future studies to definitively link this variant to endothelial dysfunction.

## Conclusion

In conclusion, the primary contribution of this work is the establishment of a novel genetic baseline for the *NOS3* rs1799983 variant across isolated, critically underrepresented archipelagic populations in Indonesia. By mapping the allelic landscape of groups virtually absent from global databases such as the 1000 Genomes Project and gnomAD, this study addresses a significant gap in global genomic representation. Secondarily, an initial unadjusted association with hypertension was observed in the Morotai subpopulation. While this unadjusted finding did not persist following rigorous correction for multiple testing, an independent association emerged after multivariate adjustment for clinical covariates, surviving FDR correction (adjusted *p* = 0.006). Consequently, this localized association serves as a preliminary, hypothesis-generating finding that warrants targeted investigation in future longitudinal cohorts.

## Data Availability

The original contributions presented in the study are publicly available. The anonymized genotypic frequencies and subpopulation datasets can be found in the following link https://doi.org/10.6084/m9.figshare.32576259. Further inquiries can be directed to the corresponding author.
